# A Neuro-Immune Score Defines a Stromal–Neural and Immune-Segregated Microenvironment Associated with Poor Prognosis in Colorectal Cancer

**DOI:** 10.3390/ijms27167231

**Published:** 2026-08-13

**Authors:** Hui Hu, Xu Yuan, Fang Peng, Na Shen, Yanjun Lu

**Affiliations:** Department of Laboratory Medicine, Tongji Hospital, Tongji Medical College, Huazhong University of Science and Technology, Wuhan 430030, China

**Keywords:** colorectal cancer, neuro-immune score, tumor microenvironment, spatial transcriptomics, single-cell RNA sequencing

## Abstract

Colorectal cancer progression and therapeutic response are determined not only by tumor-intrinsic programs but also by neural, stromal, and immune components of the tumor microenvironment. However, biologically interpretable transcriptomic scores that jointly capture neural/stromal remodeling and immune activation remain limited. We developed a neuro-immune score (NIS), defined as the neural/stromal module score minus the immune activation module score. NIS was constructed using the combined TCGA-COAD/READ colorectal cancer cohort and externally evaluated in independent Gene Expression Omnibus (GEO) datasets. Single-cell RNA sequencing, focused ligand–receptor analysis, and spatial transcriptomics were further integrated to characterize the cellular origins, spatial organization, and potential mechanisms underlying NIS-associated biology. A high NIS (NIS-high) was associated with adverse prognosis in bulk transcriptomic cohorts. External validation demonstrated that a high NIS was significantly associated with worse disease-free survival/relapse-free survival (DFS/RFS) in GSE39582 and worse overall survival in GSE17536. A multi-cohort meta-analysis further supported a consistent association between NIS-high and poor clinical outcomes. Single-cell analysis localized the NIS-high signal mainly to glial-like cells, fibroblasts, pericytes, endothelial cells, and malignant epithelial cells, whereas CD8+ T cells and natural killer cells exhibited low NIS. Focused ligand–receptor analysis suggested that NIS-high cellular compartments may communicate with immune and tumor compartments through MIF-CD74/CXCR4, SPP1-CD44, extracellular matrix (ECM)–integrin, TGF-β, and immune checkpoint-related axes. Spatial transcriptomics further demonstrated that NIS-high regions were enriched in stromal, neural/glial-like, vascular/pericyte, and tumor–stromal niches, whereas NIS-low regions were associated with immune-activated and cytotoxic T/NK cell-rich areas. NIS captures a spatially organized state of the colorectal cancer microenvironment characterized by neural/stromal remodeling, activation of ECM and vascular/pericyte niches, and relatively reduced or spatially segregated immune activation. NIS may serve as a biologically interpretable microenvironment stratification score associated with adverse outcomes. It also provides a framework for future studies targeting stromal remodeling, myeloid-mediated immune regulation, neural-associated signaling, and antitumor immunity.

## 1. Introduction

Colorectal cancer (CRC) is a globally prevalent malignant gastrointestinal tumor whose incidence, progression, and clinical outcomes are influenced by genetic susceptibility, inflammation, diet, medication exposure, and broader host–environmental factors [1,2]. Although the American Joint Committee on Cancer (AJCC) staging system remains central to clinical decision-making, patients within the same stage can experience markedly different outcomes [3,4,5]. This heterogeneity underscores the need for biologically interpretable biomarkers that refine risk stratification beyond conventional clinicopathological variables.

Growing evidence highlights the crucial biological and pathological roles of interactions between the nervous system and cancer [6,7]. Both the central nervous system (CNS) and the peripheral nervous system (PNS) are implicated in various cancer types throughout the body [8]. The nervous system plays a critical role in regulating immune cells by controlling their activation, proliferation, and distribution, thereby influencing normal immune function [9]. Moreover, the nervous system can directly influence immune cells within the tumor microenvironment (TME), significantly impacting cancer progression [10]. For example, peripheral nerve fibers release calcitonin gene-related peptide, which may reduce effector T lymphocytes [11], and Schwann cells can promote M2-polarized macrophages and accelerate tumor growth [12]. Neural infiltration in the TME may also influence the efficacy of immune checkpoint inhibitors. It is well established that the gastrointestinal tract possesses its own intrinsic nervous system—the enteric nervous system [13]. A previous study found that enteric serotonergic neurons release 5-hydroxytryptamine, which binds to its receptors and activates the Wnt/β-catenin signaling pathway, thereby driving the self-renewal of CRC stem cells [14]. Neural involvement plays a crucial role in colorectal cancer progression as the colon is richly innervated by autonomic and enteric nerves. Higher perineural invasion (PNI), defined as the invasion of cancer cells around nerve fibers, and higher normalized nerve density (NND) are critical factors influencing patient survival [15]. Both PNI and NND have been shown to predict shorter disease-free survival (DFS) and overall survival (OS). PNI has been proposed as a novel biological feature, and studies report that its incidence in gastrointestinal tumors ranges from 13% to 39%, with an average detection rate of 26% [16,17,18]. Exploring the role of PNI in colorectal cancer may help predict invasive and metastatic behavior and guide more precise surgical treatment [19]. Taken together, these findings suggest that developing biomarkers and therapies targeting the nervous system in CRC represents a promising direction for further investigation.

We hypothesized that the balance between neural–stromal activity and antitumor immune activity can be captured by an interpretable transcriptomic neuro-immune score (NIS). We evaluated the association of NIS with survival in the TCGA-CRC cohort, validated its prognostic direction in independent GEO cohorts, and localized its cellular and spatial basis using single-cell RNA-seq and spatial transcriptomics. This study characterizes the neural/stromal–immune balance in CRC and evaluates its prognostic and mechanistic relevance across complementary data modalities.

## 2. Results

### 2.1. Development of the NIS and Its Analytic Outcomes in Colorectal Cancer (CRC) Based on TCGA

**Figure 1:**
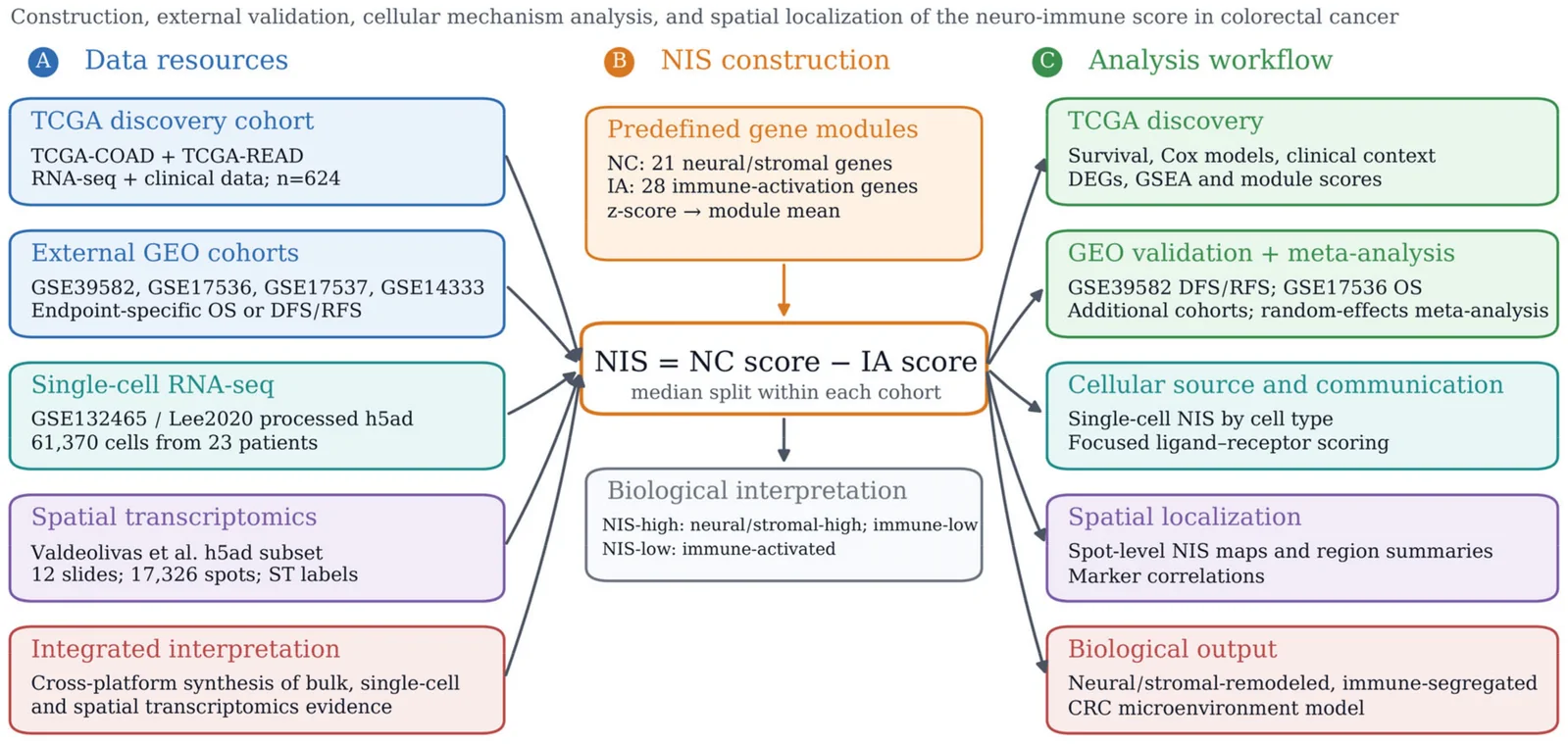
Study design and NIS analysis workflow. Data resources used in this study, including the TCGA-COAD/READ discovery cohort, external GEO validation cohorts, GSE132465 single-cell RNA-seq data, and the locally available processed Valdeolivas colorectal cancer spatial transcriptomics subset. Construction of the neuro-immune score (NIS). NIS was calculated as the neural/stromal component score minus the immune activation component score using predefined gene modules, followed by cohort-specific median stratification into NIS-high and NIS-low groups. Integrated analysis workflow, including TCGA discovery analysis, external GEO validation and meta-analysis, single-cell localization and ligand–receptor analysis, spatial transcriptomic localization, marker correlation analysis, and cross-platform biological interpretation. ST, spatial transcriptomics; OS, overall survival; DFS/RFS, disease-free or recurrence-free survival.

Figure 1 illustrates the integrated study framework for NIS construction, validation, and biological interpretation across bulk transcriptomic, single-cell, and spatial transcriptomic datasets in colorectal cancer. The overall dataset roles are summarized in Table 1. We first constructed a neuro-immune score (NIS) for colorectal cancer based on predefined neural component (NC) and immune activation (IA) gene modules. The NC module comprised 21 genes associated with neural crest/Schwann cell-like phenotypes, neurotrophic factors, axon guidance, and nerve-related stromal remodeling; the IA module included 28 genes reflecting T/NK cell cytotoxic activity, antigen presentation, T-cell markers, and immune checkpoint activation (Table S1). After log_2_(FPKM + 1) transformation of each gene in the TCGA-CRC expression matrix, gene-level z-score normalization was performed across all TCGA-CRC samples. The mean z-scores of the NC (z_NC) and IA (z_IA) modules were then calculated separately, and NIS was defined as NIS = mean(z_NC) − mean(z_IA). Additional quality-control analyses confirmed the composition of the TCGA-CRC discovery cohort, the case distribution, the opposing contributions of neural/stromal and immune activation components to NIS, and the overall balance of major clinical variables between NIS groups (Figure S1). Thus, a higher NIS indicates a tumor transcriptomic state with relatively enhanced neural-associated components and reduced immune-activation components.

**Figure 2:**
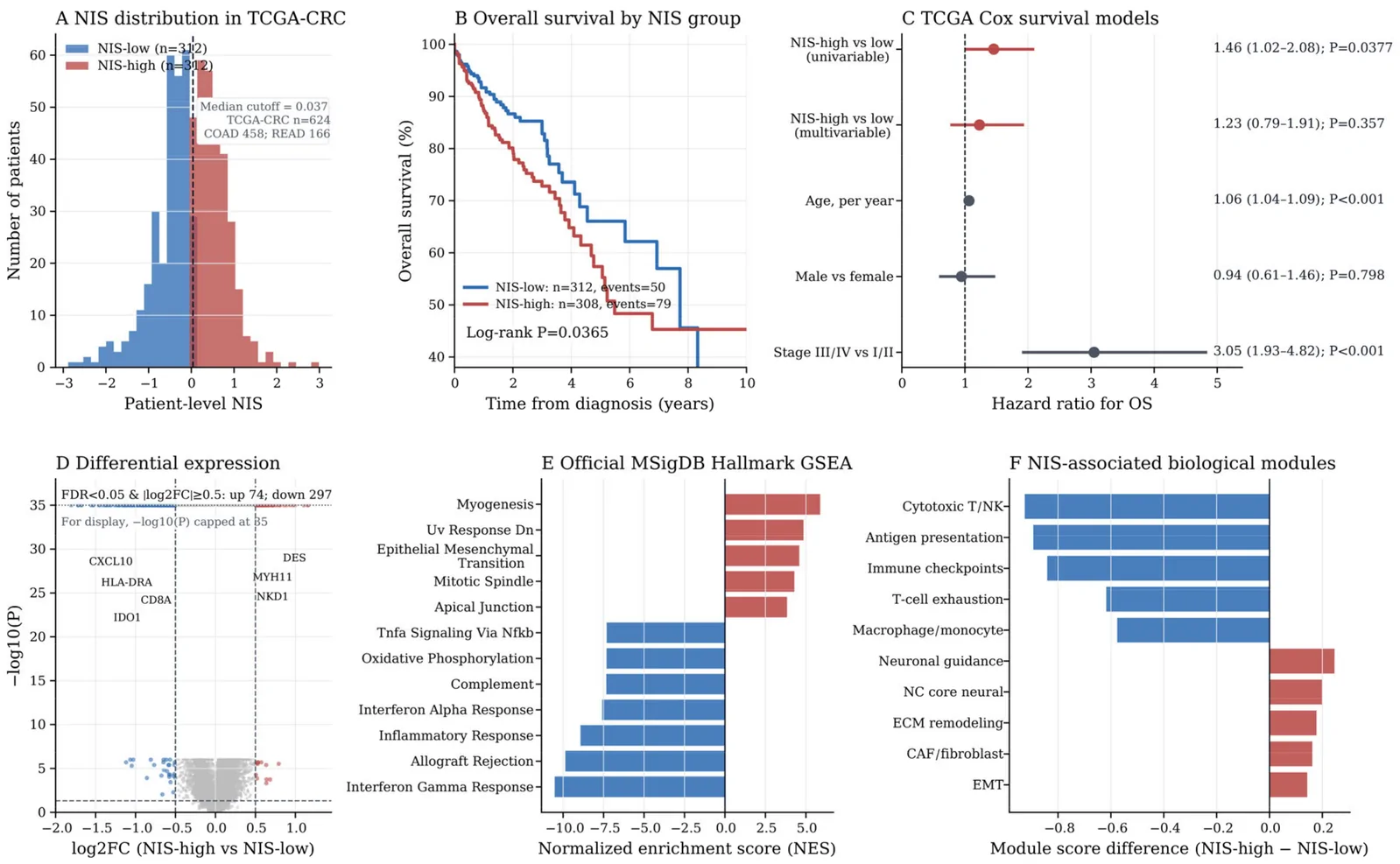
Integrated TCGA analysis of NIS in colorectal cancer. (**A**). Distribution of patient-level NIS in the combined TCGA-COAD/READ colorectal cancer cohort. The cohort median was used to define NIS-low and NIS-high groups. (**B**). Kaplan–Meier overall-survival curves comparing NIS-high and NIS-low patients in TCGA-CRC. (**C**). Cox proportional-hazards forest plot evaluating NIS and selected clinical covariates; the multivariable model adjusted for age, sex, and pathological stage; complete-case *n* = 501. (**D**). Volcano plot of differential gene expression between NIS-high and NIS-low tumors. (**E**). MSigDB Hallmark preranked gene-set enrichment analysis showing enrichment of neural/stromal remodeling programs and depletion of interferon, inflammatory, and immune activation programs in NIS-high tumors. Blue: depleted in NIS-high/enriched in NIS-low; red: enriched in NIS-high. (**F**). Custom biological module analysis showing reduced cytotoxic, antigen-presentation, and immune checkpoint modules but increased neural guidance, neural component core, and stromal remodeling modules in NIS-high tumors; the inset summarizes representative immune checkpoint gene shifts.

In the combined CRC cohort of TCGA-COAD and TCGA-READ, 624 patients were included in the patient-level NIS analysis, comprising 458 cases from TCGA-COAD and 166 from TCGA-READ (Table S2). All predefined NIS genes were detectable in the expression matrix (NC 21/21, IA 28/28). Using the median patient-level NIS of 0.037130 across the entire TCGA-CRC cohort as the cutoff, patients were divided into NIS-low (*n* = 312) and NIS-high (*n* = 312) groups (Figure 2A).

Kaplan–Meier analysis showed that the NIS-high group had significantly worse overall survival than the NIS-low group (log-rank *p* = 0.0365), with 79 events in 308 patients versus 50 events in 312 patients (Figure 2B). The survival curves remained clearly separated throughout the follow-up period, indicating a sustained association between elevated NIS and increased mortality risk in the TCGA-CRC cohort.

Univariable Cox regression analyses revealed that the dichotomized NIS (high vs. low) was significantly associated with worse overall survival (OS) in the TCGA-CRC cohort (HR 1.46, 95% CI 1.02–2.08, *p* = 0.0377). However, when assessed as a continuous variable, the NIS did not reach statistical significance (HR 1.24, 95% CI 0.97–1.60, *p* = 0.090) (Table 2 and Table S3). Among the clinical covariates, advanced pathological stage (III/IV vs. I/II) emerged as the strongest prognostic factor (HR 3.05, 95% CI 1.93–4.82, *p* < 0.001), along with age (HR 1.06 per year, 95% CI 1.04–1.09, *p* < 0.001). In contrast, sex (male vs. female, *p* = 0.798) and tumor origin (TCGA-READ vs. COAD, *p* = 0.265) showed no significant prognostic impact (Table 2 and Table S3).

In the multivariable model adjusted for age, sex, and pathological stage, the association between NIS-high and worse OS remained directionally consistent but lost statistical significance (HR 1.23, 95% CI 0.79–1.91, *p* = 0.357) (Figure 2C). This attenuation suggests that the prognostic effect of the NIS classification partly overlaps with established clinicopathological factors, particularly tumor stage and age, and that NIS was not an independent OS predictor in the adjusted TCGA model.

Sensitivity analyses yielded consistent results. When modeled as a continuous variable, NIS showed a borderline association with overall survival in univariable analysis (per 1 SD increase: HR = 1.17, 95% CI: 0.97–1.41; *p* = 0.092), but not after multivariable adjustment (adjusted HR = 1.07; *p* = 0.54). Alternative dichotomization thresholds, including the upper tertile and upper quartile, produced associations in the same direction but did not improve adjusted prognostic performance. Moreover, adding NIS to the model containing age, sex, and stage resulted in negligible changes in discrimination (ΔC-index ≤ 0.002), no significant improvement by likelihood-ratio testing for continuous NIS, and only modest changes in time-dependent AUC and continuous NRI. Collectively, these findings support interpreting NIS as a risk-associated feature of the tumor microenvironment rather than as an independently validated prognostic model (Table S12).

### 2.2. NIS Reveals Neuro-Immune Phenotypic Features in TCGA CRC

To elucidate the transcriptomic features associated with the NIS-high phenotype, we compared gene expression profiles between NIS-high and NIS-low tumors in the patient-level TCGA-CRC cohort. A total of 19,945 protein-coding gene symbols were examined. At a threshold of FDR < 0.05 and |log_2_ fold change | ≥ 0.5, we identified 371 significantly differentially expressed genes, of which 74 were upregulated and 297 were downregulated in the NIS-high group (Figure 2D). The downregulated genes in the NIS-high group were predominantly related to immune activation, including JCHAIN, CXCL10, CXCL9, HLA-DRA, GZMA, CCL5, IDO1, NKG7, CD3D, and CD8A, whereas the upregulated genes included NKD1, SYT7, PALD1, MEX3A, PMEPA1, MYH11, SYNM, RNF43, HSPB7, and ALKAL1 (Table S4).

GO and KEGG enrichment analyses further supported that NIS-high tumors exhibit a state of low immune activation and pronounced neural/stromal remodeling. The downregulated genes in the NIS-high group were enriched in processes related to immune response, interferon/chemokine signaling, antigen presentation, and T/NK cell activation; in contrast, the upregulated genes were predominantly associated with WNT/developmental signaling, smooth muscle/myofibroblast-like phenotypes, ECM/cell adhesion, and neural-related pathways. Consistently, KEGG enrichment indicated that downregulated genes were mainly involved in immune–inflammatory pathways, whereas upregulated genes pointed to stromal remodeling and development/neural-associated transcriptional programs (Table S5).

Preranked GSEA using the MSigDB Hallmark gene sets revealed that the NIS-high direction was significantly enriched for pathways such as myogenesis, epithelial–mesenchymal transition, Hedgehog signaling, Wnt/β-catenin signaling, angiogenesis, TGF-β signaling, and Notch signaling. Conversely, interferon-gamma response, allograft rejection, inflammatory response, interferon-alpha response, complement, TNFα/NF-κB, IL2/STAT5, IL6/JAK/STAT3, and antigen/immune-related signatures were significantly negatively enriched in the NIS-high phenotype (Figure 2E).

Custom module score analyses (GSVA-like) yielded results consistent with the DEG and GSEA findings: the NIS-high group exhibited significantly lower scores for immune-related modules such as cytotoxic_T_NK, Antigen_presentation, Immune_checkpoint_inhibitory, T_cell_exhaustion, and Macrophage_monocyte, whereas scores for neuro-stromal modules including Neuronal_guidance, NC_core_neural, ECM_remodeling, CAF_fibroblast, and EMT were elevated in the NIS-high group. At the individual immune checkpoint gene level, IDO1, LAG3, HAVCR2, CD274, PDCD1, and CTLA4 were all markedly downregulated in the NIS-high group (Figure 2F and Figure S2).

Collectively, the NIS-high phenotype does not merely represent an increase in neural-related genes, but rather reflects a composite transcriptomic state characterized by enhanced neural/stromal remodeling coupled with reduced immune inflammation and antigen presentation, which may be summarized as a neural-enriched/immune-cold phenotype. These findings provide a mechanistic basis for the subsequent GEO validation and for single-cell and spatial localization analyses.

### 2.3. External Validation of the Prognostic Value of NIS in Independent GEO Cohorts

**Figure 3:**
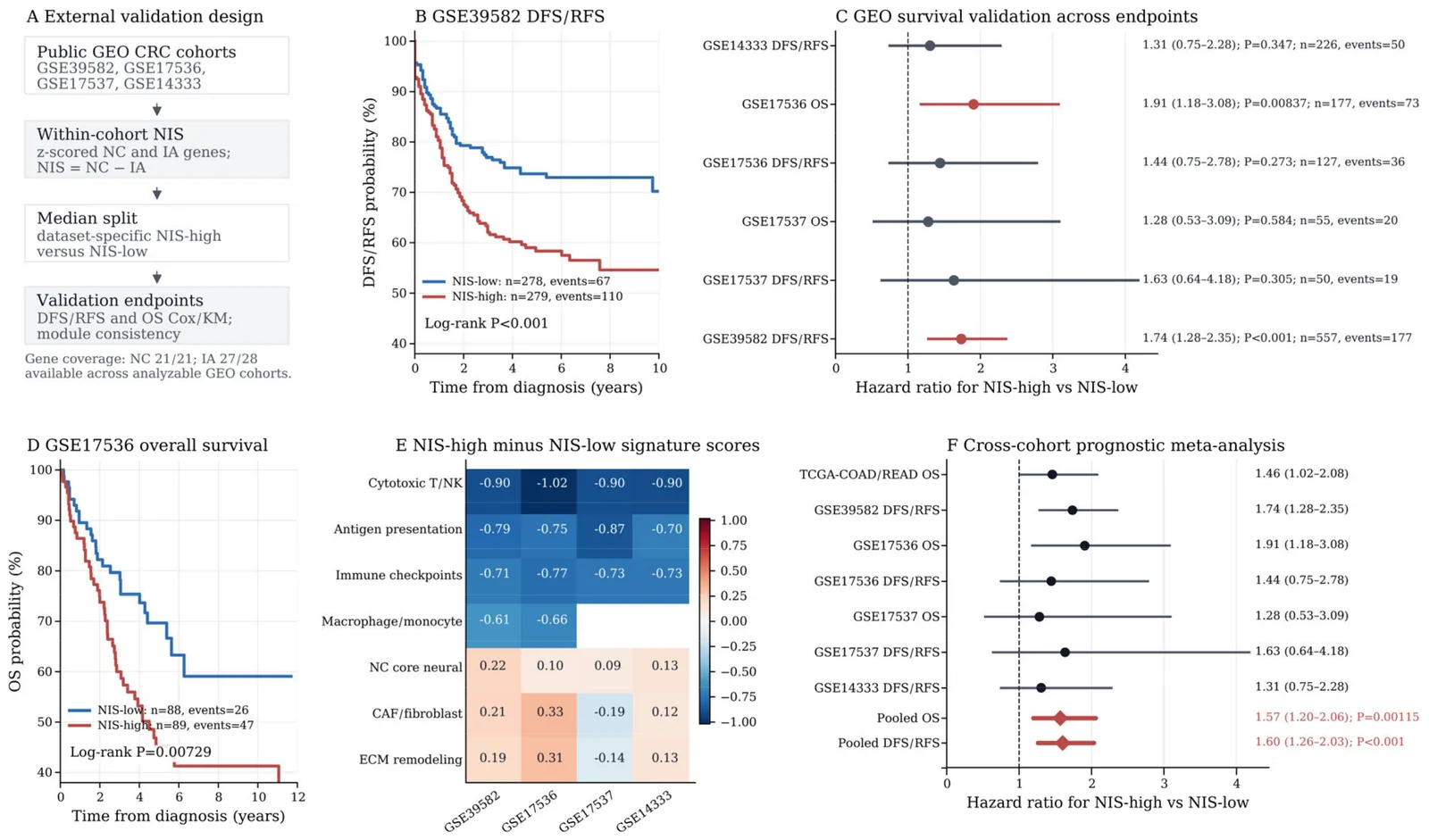
External GEO validation of the prognostic and biological relevance of NIS. (**A**). External validation workflow. NIS was recalculated within each GEO colorectal cancer cohort using cohort-level z-scored neural/stromal and immune-activation genes and dichotomized at the cohort-specific median. (**B**). Kaplan–Meier DFS/RFS curves in the primary external validation cohort GSE39582. (**C**). Univariable Cox forest plot across analyzable GEO survival endpoints. (**D**). Kaplan–Meier overall-survival curves in GSE17536. (**E**). High-minus-low differences in predefined biological signature scores across GEO cohorts, showing reduced immune-activation modules and increased neural/stromal remodeling modules in NIS-high tumors. Blue: immune activation lower in NIS-high; red: neural/stromal modules higher in NIS-high. (**F**). Random-effects meta-analysis integrating TCGA and GEO prognostic endpoints, supporting the overall adverse prognostic association of NIS-high status across cohorts.

To evaluate the robustness and generalizability of the neuro-immune score (NIS), we performed external validation in multiple independent colorectal cancer cohorts from GEO. Gene expression data were obtained from the curatedCRCData resource whenever available, using uniformly processed fRMA gene-level ExpressionSet objects to reduce variability arising from probe annotation and preprocessing differences across platforms. The overall validation design is shown in Figure 3A.

GSE39582 was used as the primary external validation cohort for recurrence-related outcomes. A total of 557 patients had evaluable DFS or RFS data, with 177 events.

Kaplan–Meier analysis showed that patients in the NIS-high group had significantly worse DFS/RFS than those in the NIS-low group (log-rank *p* = 0.00028; Figure 3B). In univariable Cox regression analysis, NIS-high was significantly associated with an increased risk of recurrence or death (HR = 1.74, 95% CI 1.28–2.35, *p* < 0.001; Figure 3C). After adjustment for age, sex, and pathological stage, NIS-high remained associated with worse DFS/RFS (adjusted HR = 1.74, 95% CI 1.28–2.35, *p* = 0.00038; Table S6). These findings indicate that the prognostic value of NIS was successfully reproduced in an independent large GEO cohort and was not fully explained by standard clinicopathological variables.

The prognostic value of NIS was further evaluated in three additional independent GEO datasets. In GSE17536 (*n* = 177, 73 OS events), NIS-high was significantly associated with worse overall survival in both univariable (HR = 1.91, 95% CI 1.18–3.08, *p* = 0.008) and multivariable (HR = 1.95, *p* = 0.016) analyses (Figure 3C,D, Table S6). For DFS/RFS (*n* = 127, 36 events), the association was directionally consistent but not statistically significant (HR = 1.44, *p* = 0.273). In GSE17537 and GSE14333, NIS-high showed directionally consistent but non-significant associations with adverse outcomes. In GSE17537, the HRs were 1.28 for OS and 1.63 for DFS/RFS. In GSE14333, OS was unavailable or insufficient for analysis, whereas DFS/RFS showed a directionally consistent association (HR = 1.31) (Figure 3C, Table S6). These results should be interpreted cautiously due to limited sample sizes, event numbers, endpoint availability, and covariate completeness.

To evaluate the prognostic effect of NIS across cohorts, we performed separate random-effects meta-analyses for OS and DFS/RFS using univariable Cox regression estimates from TCGA and GEO validation cohorts. For OS, three cohort-level estimates were included: TCGA-COAD/READ, GSE17536, and GSE17537. The NIS-high was significantly associated with worse OS (pooled HR = 1.57, 95% CI 1.20–2.06, *p* = 0.001; Figure 3F). For DFS/RFS, four cohort-level estimates were included: GSE39582, GSE17536, GSE17537, and GSE14333. NIS-high was likewise associated with poorer DFS/RFS (pooled HR = 1.60, 95% CI 1.26–2.03, *p* < 0.001; Figure 3F). Collectively, these endpoint-specific analyses consistently associated high NIS with adverse clinical outcomes across cohorts.

To assess whether the biological features associated with NIS in TCGA were reproducible in independent datasets, we evaluated a panel of predefined signatures in external GEO cohorts, covering neural/stromal programs, cytotoxic T/NK cell activity, antigen presentation, immune checkpoint regulation, CAF/fibroblast markers, extracellular matrix remodeling, EMT, and TGF-β-related pathways. Across the evaluated external cohorts, NIS-high tumors generally exhibited elevated neural/stromal, CAF, ECM, and mesenchymal-related signatures, together with reduced cytotoxic immune activity, antigen presentation, and immune checkpoint-associated signatures (Figure 3E). This pattern closely mirrored the TCGA findings and further supports the interpretation that the NIS-high phenotype represents a tumor microenvironment characterized by augmented neural/stromal remodeling and diminished antitumor immune activation. To assess the robustness of external validation, we further examined NIS gene coverage, endpoint-specific sample and event counts, and cohort-level signature concordance. These analyses supported adequate cross-cohort gene coverage and broadly concordant biological and prognostic patterns despite differences in cohort size and endpoint definition (Figure S3).

### 2.4. Comparison of NIS with Established Transcriptomic Classifiers

To determine whether NIS provides prognostic information beyond existing transcriptomic scores, we compared its performance with immune, stromal, EMT, CAF/ECM, and cytotoxic T/NK signatures, as well as exploratory CMS-like subtypes, using TCGA-CRC data. Median-dichotomized NIS was associated with poorer overall survival in univariable analysis (HR = 1.45, 95% CI: 1.01–2.06, *p* = 0.042), although this association was attenuated after adjustment for age, sex and stage (HR = 1.22, 95% CI: 0.79–1.90, *p* = 0.37). Although NIS had the numerically highest adjusted C-index among the median-dichotomized transcriptomic scores (0.738), its improvement over the clinical model was small (ΔC-index = 0.0018), indicating prognostic performance broadly comparable to, rather than consistently superior to, the other evaluated scores. NIS showed weak positive correlations with stromal, EMT and CAF/ECM scores (Spearman rho = 0.156–0.165) but strong inverse correlations with immune and cytotoxic T/NK scores (rho = −0.785 and −0.749, respectively) (Figure S4A). NIS was highest in CMS4-like mesenchymal tumors and lowest in CMS1-like immune tumors (Kruskal–Wallis *p* = 2.11 × 10^−35^) (Figure S4B), while stromal-high/immune-low tumors had substantially higher NIS than other tumors. Within the CMS4-like subgroup, higher NIS showed a non-significant trend toward poorer survival (HR = 1.77, 95% CI: 0.96–3.28, *p* = 0.069). Collectively, these findings do not demonstrate universal prognostic superiority of NIS but support its complementary biological interpretability as a score that integrates neural/stromal remodeling with reduced immune activation (Table S11).

### 2.5. Single-Cell Analysis Identifies the Cellular and Communication Basis of NIS

To define the cellular origin of NIS, we analyzed the GSE132465 colorectal cancer single-cell RNA-seq dataset, including 61,370 cells from 23 patients. NIS was reconstructed at single-cell resolution as the difference between the neural/stromal component score and the immune activation component score, with high gene coverage for both modules. Supplementary Figure S6 shows the localization of the neuro-immune score (NIS), NC score, IA score, and selected NIS-high or IA-high cellular states at single-cell resolution in the GSE132465 colorectal cancer dataset.

**Figure 4:**
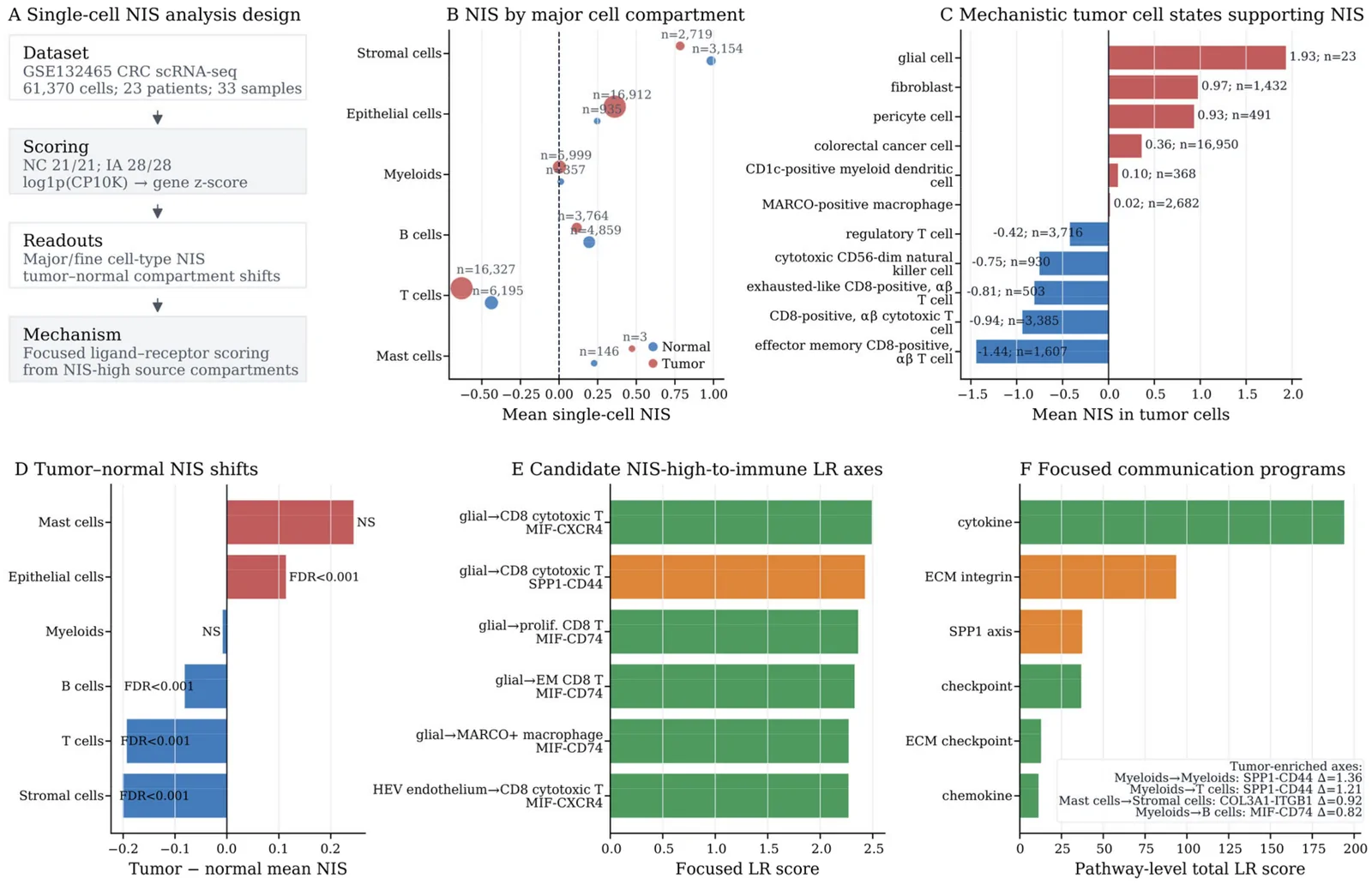
Single-cell localization and candidate communication mechanisms of NIS. (**A**). Analysis workflow for the GSE132465/Lee2020 colorectal cancer single-cell RNA-seq dataset, including cell-level NIS calculation, compartment-level comparison, tumor–normal contrast, and focused ligand–receptor analysis. (**B**). Major-compartment NIS showing higher NIS in stromal and epithelial compartments and lower or negative NIS in T-cell compartments. Point size denotes number of cells; red = tumor, blue = normal. (**C**). Fine cell-state ranking in tumor samples, highlighting glial cells, fibroblasts, pericytes, and colorectal cancer cells as NIS-high compartments, whereas cytotoxic, effector-memory, exhausted CD8 T cells and NK cells represent NIS-low immune activation compartments. (**D**). Tumor-versus-normal shifts in mean NIS by major cell type. (**E**). Candidate ligand–receptor axes from NIS-high source compartments to immune target populations. Hypothesis-driven LR score = mean ligand in source × mean receptor in target. (**F**). Pathway-level focused communication programs.

NIS was predominantly enriched in non-immune compartments of the tumor microenvironment. In tumor tissue, stromal cells exhibited the highest mean NIS (0.784), followed by epithelial cells (0.361), whereas T cells showed markedly negative NIS (−0.631) (Figure 4B, Table S7). Fine-grained cell-type analysis further localized high NIS to glial cells, fibroblasts, pericytes, high endothelial venule (HEV) endothelial cells, and colorectal cancer cells. In contrast, cytotoxic immune populations, including effector memory CD8+ T cells, cytotoxic CD8+ T cells, exhausted-like CD8+ T cells, and NK cells, displayed strongly negative NIS values (Figure 4C).

Comparison between tumor and normal tissues revealed that tumor epithelial cells had higher NIS than normal epithelial cells, whereas tumor-infiltrating T cells had lower NIS than normal T cells (Figure 4D). These findings suggest that NIS-high tumors are characterized by enrichment of neural/stromal, vascular, fibroblast, and malignant epithelial programs, together with low or negative NIS values in cytotoxic T/NK compartments, reflecting relatively high immune-activation module activity in these immune states. Focused ligand–receptor analysis further suggested that NIS-high compartments may communicate with immune and tumor compartments through MIF–CD74/CXCR4, SPP1–CD44, ECM–integrin, immune checkpoint, TGF-β, chemokine, vascular, and neural guidance-related axes (Figure 4E). At the pathway level, the strongest signals involved cytokine/myeloid immune regulation, ECM–integrin stromal barrier signaling, SPP1-mediated macrophage–stromal interaction, and immune checkpoint signaling (Figure 4F and Figure S5).

Together, these single-cell results support a compartmental model in which high bulk NIS reflects coordinated enrichment of neural/stromal and malignant epithelial programs, stromal and vascular remodeling, and altered immune communication. This provides a cellular and mechanistic explanation for the adverse prognostic significance of NIS observed in bulk cohorts.

### 2.6. Spatial Transcriptomics Validates the Tissue Localization of NIS Biology

**Figure 5:**
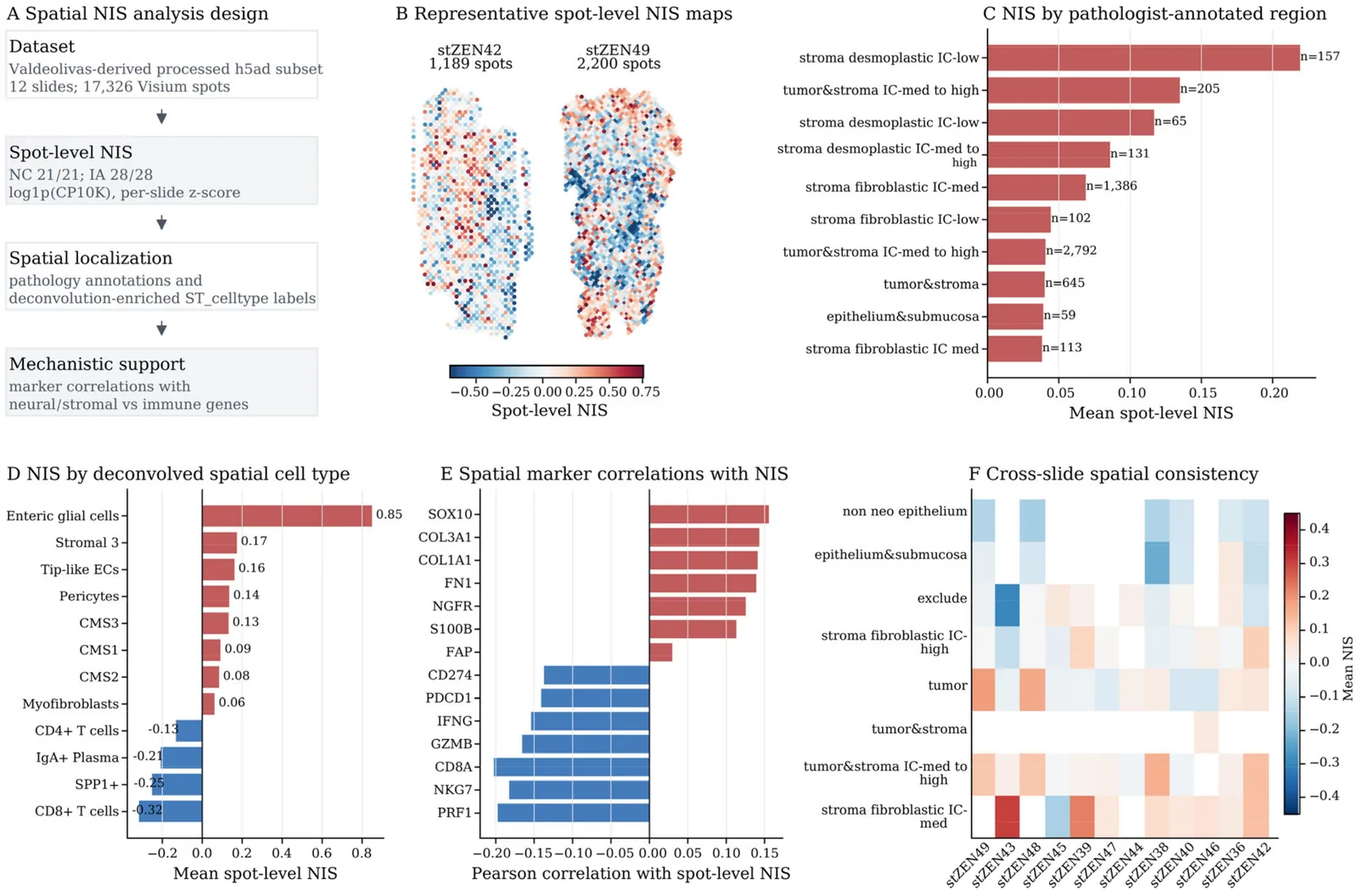
Spatial transcriptomic localization of NIS in colorectal cancer tissue. (**A**). Spatial transcriptomics analysis workflow using the locally available processed h5ad subset derived from the Valdeolivas colorectal cancer spatial transcriptomics dataset, including 12 slides and 17,326 Visium spots. Spot-level NIS was recalculated from the expression matrix within each slide. (**B**). Representative spatial maps showing the tissue-level distribution of spot-level NIS. (**C**). NIS ranking across pathologist-annotated tissue regions, showing enrichment of higher NIS in stromal and tumor–stromal regions. (**D**). NIS ranking across deconvolution-enriched spatial cell-type labels, highlighting higher NIS in enteric glial, stromal/myofibroblast, endothelial, and pericyte-associated spatial contexts. (**E**). Correlations between spot-level NIS and selected neural/stromal, extracellular-matrix, cytotoxic immune, antigen-presentation, and immune checkpoint markers. Positive: neural/stromal markers; negative: cytotoxic/checkpoint/IFN markers. (**F**). Cross-slide heatmap of mean NIS across recurrent pathology annotations. Rows show recurrent pathology annotations; columns are analyzed slides.

To evaluate the spatial organization of NIS-related biology, we analyzed a locally available processed h5ad subset derived from the Valdeolivas et al. CRC spatial transcriptomics dataset, comprising 12 spatial transcriptomics slides and 17,326 spots (https://zenodo.org/records/7760264 (accessed on 10 January 2026)). NIS was calculated at the spot level using the same definition as in the bulk and single-cell analyses: NIS = NC_score − IA_score. Gene coverage was high across slides, with 21/21 NC genes and 28/28 IA genes available (Figure 5A).

Spatial region analysis showed that high NIS was mainly localized to stromal and tumor–stromal regions (Figure 5B). Based on pathologist annotation, the highest mean NIS values were observed in desmoplastic stroma with low immune-cell infiltration (mean NIS = 0.219), tumor-stroma regions with medium-to-high immune-cell infiltration (mean NIS = 0.135), and selected immune-cell aggregate regions in the muscularis or stroma (mean NIS = 0.133) (Figure 5C). In contrast, several immune-cell aggregate regions in submucosa or stroma/muscularis exhibited low or negative NIS, consistent with increased IA module activity.

Deconvolved spatial cell-type labels further supported this localization pattern. The highest NIS was observed in enteric glial cells (mean NIS = 0.850), followed by Stromal 1 (0.359), Stromal 3 (0.173), tip-like endothelial cells (0.161), and pericytes (0.135) (Figure 5D). These spatial findings were consistent with the single-cell analysis, in which glial, fibroblast, pericyte, and endothelial compartments showed high NIS. Conversely, CD8+ T cells, NK cells, T helper 17 cells, and SPP1+ immune-associated regions showed low or negative NIS values, consistent with the IA-high immune compartments identified at single-cell resolution.

Marker correlation analysis at the spot level demonstrated positive correlations between NIS and neural/stromal or ECM-related markers, including SOX10, COL3A1, COL1A1, FN1, NGFR, and S100B. In contrast, NIS was negatively correlated with immune activation and antigen-presentation markers, including CD8A, GZMB, and HLA-DRA (Figure 5E, Table S8). To further assess the reproducibility of these spatial patterns across individual slides, we summarized mean spot-level NIS values for recurrent pathology annotations across the analyzed spatial transcriptomics slides. Although inter-slide heterogeneity was observed, NIS-positive signals were repeatedly detected in stromal, tumor–stromal, and immune-cell-intermediate spatial regions, whereas non-neoplastic epithelial or excluded regions generally showed lower or negative NIS values (Figure 5F). This cross-slide pattern indicates that NIS enrichment is not restricted to a single representative section, but rather reflects a recurrent spatial organization of neural/stromal-remodeled tumor microenvironment niches.

Overall, the spatial transcriptomics analysis validated the tissue-level organization of NIS biology. High NIS was not randomly distributed but localized to stromal, neural/glial-like, vascular/pericyte, and tumor–stromal niches, whereas low NIS was associated with immune-activated or immune-cell-rich regions. These spatial findings reinforce the bulk prognostic and single-cell mechanistic analyses, supporting the characterization of NIS-high as a spatially organized tumor microenvironment state defined by neural/stromal remodeling and relatively reduced or spatially segregated immune activation.

## 3. Discussion

In this study, we developed and evaluated a neuro-immune score (NIS) to characterize a clinically relevant tumor microenvironment state in colorectal cancer (CRC). NIS captures the balance between neural/stromal programs and immune activation programs, defined as the neural/stromal component minus the immune activation component. Across bulk transcriptomic cohorts, external GEO validation datasets, single-cell RNA-seq, focused ligand–receptor analysis, and spatial transcriptomics, our findings consistently indicate that high NIS identifies a CRC microenvironment enriched for neural/stromal remodeling, extracellular matrix organization, vascular/pericyte-associated niches, and relatively reduced or spatially segregated cytotoxic immune activity.

An association between NIS and adverse outcomes was observed in both discovery and validation analyses. In independent GEO cohorts, NIS-high tumors were associated with poorer clinical outcomes, most notably in GSE39582 for DFS/RFS and in GSE17536 for overall survival. Although some smaller cohorts showed only directionally consistent, non-significant associations, separate random-effects meta-analyses for OS and DFS/RFS both demonstrated a consistent adverse prognostic direction (pooled HR = 1.57 and 1.60, respectively). These findings suggest that NIS may capture a reproducible biological state rather than a dataset-specific statistical pattern.

A key strength of this study is that NIS was not only associated with prognosis in bulk cohorts but was also biologically interpretable at single-cell and spatial levels. Single-cell analysis of GSE132465 showed that NIS-high signals were predominantly enriched in stromal cells, glial-like cells, fibroblasts, pericytes, endothelial cells, and malignant epithelial cells, whereas cytotoxic immune populations (CD8+ T-cell subsets and NK cells) displayed strongly negative NIS values, reflecting high immune-activation module expression. Thus, NIS is not a tumor cell-intrinsic score; rather, it reflects the relative contributions of neural/stromal and immune compartments within the tumor ecosystem.

Spatial transcriptomics further reinforced this interpretation. High NIS localized to desmoplastic stromal, tumor–stromal, enteric glial, endothelial, and pericyte-associated niches, whereas low NIS was associated with immune-activated or immune-cell-rich regions. Spot-level correlations showed that NIS was positively associated with neural/stromal and ECM-related markers such as SOX10, COL1A1, COL3A1, FN1, NGFR, and S100B, but negatively associated with immune activation markers such as CD3D, CD8A, PRF1, NKG7, HLA-DRA, and GZMB. These results provide spatial evidence that the NIS-high phenotype corresponds to organized tissue niches with stromal and neural-like features.

Our findings are consistent with, but not identical to, established CRC molecular frameworks. The consensus molecular subtype (CMS) classification defines CMS4 tumors as mesenchymal, stromal-rich, TGF-β-active, angiogenic, and poor-prognosis [20]. NIS-high tumors share several of these features, including stromal enrichment, ECM remodeling, vascular/pericyte involvement, and adverse prognosis. However, NIS differs from CMS4 by explicitly incorporating a neural/stromal-versus-immune activation balance. The prominent NIS signal in enteric glial and glial-like compartments suggests that NIS may capture a neural-microenvironmental dimension that is not fully represented by conventional CMS classification. Comparison with immune, stromal, EMT, and CAF/ECM transcriptomic scores showed that NIS did not universally outperform these signatures; its prognostic performance was broadly comparable, and the incremental C-index improvement over clinical covariates was modest. The main value of NIS is complementary biological interpretability: it summarizes the balance between neural/stromal remodeling and reduced immune activation and identifies a stromal-high/immune-low state relevant to CRC tumor ecology. Given that several NC genes are canonical stromal or ECM-associated genes, NIS is expected to overlap with stromal biology and may be interpreted as complementary to, rather than a replacement for, established frameworks such as CMS4, ESTIMATE, and EMT signatures. Future prospective cohorts with formal CMS/ESTIMATE classification and treatment annotation will be needed to test whether NIS adds clinically actionable predictive value beyond these existing biomarkers.

Consistent with recent studies highlighting the role of CAFs, ECM remodeling, and macrophage–stromal crosstalk in CRC progression, FAP+ fibroblasts and SPP1+ macrophages co-localize in desmoplastic regions, correlate with reduced lymphocyte infiltration, and predict poor outcomes [21]. Other studies have identified SPP1+ macrophages as clinically relevant immunosuppressive populations in CRC, associated with EMT, angiogenesis, hypoxia, metastasis, and therapy resistance [22,23]. In our focused ligand–receptor analysis, NIS-high source compartments showed candidate interactions with immune and tumor compartments through MIF–CD74/CXCR4, SPP1–CD44, ECM–integrin, checkpoint, TGF-β, chemokine, vascular, and neural guidance-related axes. These results do not prove causal signaling but provide plausible mechanistic links between NIS-high stromal/neural niches and immune modulation.

Several mechanisms may explain why NIS-high tumors are associated with poor prognosis. First, neural and glial-like programs may contribute to tumor progression via neurotrophic factors, axon-guidance molecules, and neuro-immune crosstalk. Emerging evidence indicates that neural signaling can influence CRC growth, invasion, metastasis, and therapeutic resistance, and perineural invasion is an adverse pathological feature in CRC [24]. The enrichment of NIS in glial-like cells and enteric glial spatial labels supports the possibility that neural-associated niches contribute to aggressive tumor biology. Second, stromal and ECM remodeling may promote immune exclusion. CAFs, collagen deposition, fibronectin remodeling, and integrin signaling can form physical and biochemical barriers that limit cytotoxic immune cell access to tumor cells, consistent with CMS4 biology and spatial studies showing that fibroblast-rich regions structure immune infiltration patterns [25]. Our data show that NIS-high regions are enriched for stromal and ECM markers, whereas immune cytotoxic markers are inversely correlated with NIS, supporting a model in which ECM-rich niches spatially separate immune activation from malignant epithelial compartments. Third, myeloid regulation may contribute to the NIS-high state. Focused communication analysis highlighted MIF–CD74/CXCR4 and SPP1–CD44 axes, involving glial-like, stromal, endothelial, pericyte, epithelial, and myeloid compartments; these pathways have been implicated in macrophage regulation, immunosuppression, and T-cell dysfunction [26]. In addition, publicly available protein-level data provide orthogonal support for the stromal/ECM and cytotoxic immune compartments represented by NIS, though prospective validation using matched transcriptomic, histological, and spatial assays in CRC tissues is warranted. Therefore, NIS-high may reflect not only stromal abundance but also a broader immunoregulatory network involving macrophage–stromal and epithelial–immune crosstalk.

This study has potential translational implications. NIS may serve as a stratification aid to identify tumors characterized by a neural/stromal-high and immune-activation-low microenvironment, which may require therapeutic strategies beyond immune checkpoint blockade alone. Potential directions include combining immunotherapy with approaches targeting TGF-β signaling, CXCR4, SPP1/CD44, CAF/ECM remodeling, angiogenic or pericyte-associated niches, or neural-related signaling. NIS could also be integrated with established features such as MSI/MMR status, CMS, tumor budding, desmoplastic reaction, perineural invasion, and immune score to improve biological risk stratification. However, prospective, treatment-annotated cohorts are required before any claim of therapeutic stratification can be made.

Several limitations should be acknowledged. First, the study was retrospective and based on public datasets, and independent prognostic value beyond standard clinicopathological variables was not established in the TCGA cohort; prospective validation in clinically annotated cohorts is required. Second, external validation cohorts differed in sample size, endpoint definitions, follow-up duration, and covariate availability, which may explain why some datasets showed only non-significant directional trends. Third, single-cell and spatial analyses provide hypothesis-generating mechanistic evidence but do not establish causality. The ligand–receptor analysis was focused and expression-based, and should not be interpreted as proof of functional intercellular signaling. Fourth, NIS was derived from RNA expression; protein-level validation using immunohistochemistry, multiplex immunofluorescence, spatial proteomics, or RNA in situ hybridization is warranted. Finally, whether NIS predicts response to immunotherapy or stromal/neural-targeted therapies remains to be tested in treatment-annotated cohorts.

## 4. Methods

### 4.1. TCGA Cohort and Data Preprocessing

The primary transcriptomic cohort consisted of TCGA colorectal cancer samples from the TCGA-COAD and TCGA-READ projects. Processed RNA-seq expression data, sample metadata, and clinical metadata were obtained from the project-level files bulk_expression_matrix.csv, bulk_sample_metadata.csv, and bulk_clinical_metadata.csv. Gene expression values were transformed as log2(FPKM + 1). Expression samples were mapped to TCGA patient identifiers using the sample metadata. When multiple expression samples were available for the same patient, patient-level NIS and expression-derived scores were averaged. The final patient-level TCGA-CRC NIS cohort included 624 patients, comprising 458 TCGA-COAD and 166 TCGA-READ cases.

### 4.2. Construction of the Neuro-Immune Score

The neuro-immune score (NIS) was constructed from two literature-curated gene modules [27,28] that were defined a priori without outcome-driven feature selection. The neural component (NC) was defined as a neural–stromal remodeling module rather than a purely neuronal signature. Its 21 genes were grouped into five functional categories: (i) Schwann/glial-like or neural crest-associated markers (S100B, SOX10, NGFR, MPZ, PLP1), (ii) neurotrophic factors and receptors (NGF, BDNF, NTRK1, NTRK2, NTRK3, GDNF, ARTN), (iii) axon-guidance or neural-communication genes (SEMA3A, SEMA4D, SLIT2, ROBO1), (iv) neural-associated stromal/ECM remodeling genes (FN1, COL1A1, COL1A2, COL3A1), and (v) a neural/stress/metabolic-associated gene (VDAC1). COL1A1, COL1A2, COL3A1, and FN1 were retained to capture stromal/ECM remodeling associated with neural-related processes and were not interpreted as markers of neuronal identity. The immune activation (IA) module comprising 28 genes was curated to represent five immune categories: cytotoxic effector genes (GZMB, GZMA, PRF1, GNLY, NKG7, IFNG), T-cell markers (CD3D, CD3E, CD2, TRAC, CD8A, CD8B), NK/innate immune markers (KLRD1, FCGR3A), antigen-presentation genes (HLA-A, HLA-B, HLA-C, HLA-DRA, HLA-DRB1, B2M, TAP1, TAP2), and checkpoint or T-cell activation-inhibition markers (CD274, PDCD1, CTLA4, HAVCR2, LAG3, TIGIT). No outcome-driven feature selection, survival-derived weighting, or regression-based optimization was applied; each gene contributed equally after within-cohort z-score standardization. Gene-level functional annotations, inclusion rationales, and supporting references are provided in Table S1. For each gene, log2(FPKM + 1) expression was z-scored across all TCGA-CRC expression samples.

For each sample, the NC and IA module scores were calculated as the average z-score of all detected genes in the corresponding module. NIS was defined as NIS = mean(zNC genes) − mean(zIA genes). Thus, higher NIS values indicate a relative enrichment of neural-associated transcriptional programs and reduced immune-activation signals. Patients were classified as NIS-high or NIS-low using the median patient-level NIS value in the full TCGA-CRC cohort, resulting in 312 patients in each group.

### 4.3. Survival and Clinical Analyses

Overall survival (OS) time and event status were derived from the curated TCGA clinical metadata. Kaplan–Meier curves were estimated for NIS-high and NIS-low groups and compared using the log-rank test. Cox proportional-hazards models were employed to evaluate the association between NIS and OS. Univariable models were fitted for dichotomous NIS, continuous NIS, age, sex, stage, and project; a simplified multivariable model was adjusted for age, sex, and pathological stage. Pathological stage was simplified as stage I/II versus III/IV. To assess the robustness of median-based dichotomization, sensitivity analyses were performed using alternative cutoffs: upper tertile versus lower two tertiles, upper quartile versus lower three quartiles, and upper quartile versus lower quartile. Incremental prognostic value beyond the clinical base model (age, sex, stage) was assessed by likelihood-ratio tests, change in Harrell’s C-index, and approximate 3- and 5-year time-dependent AUC. Continuous net reclassification improvement (NRI) was calculated at 3 and 5 years by comparing predicted risks from the base model with and without continuous NIS; patients censored before the time point without an event were excluded from the corresponding binary risk set.

### 4.4. Differential Expression Analysis

Differential expression analysis compared NIS-high and NIS-low tumors in the patient-level TCGA-CRC cohort. For each protein-coding gene symbol, patient-level log2(FPKM + 1) values were used. Duplicate gene symbols were averaged before group comparison. The log2 fold change was defined as the difference in mean expression between NIS-high and NIS-low tumors. Welch-style z statistics and two-sided *p* values were calculated for each gene, followed by Benjamini–Hochberg false discovery rate (FDR) correction. Significant differentially expressed genes (DEGs) were defined as those with FDR < 0.05 and |log2FC| ≥ 0.5.

### 4.5. Hallmark Preranked GSEA and GSVA-like Custom Module Scoring

Official MSigDB Human Hallmark gene sets were used for preranked gene-set enrichment analysis (GSEA). The ranked list was derived from the z statistics of the TCGA NIS-high versus NIS-low differential expression analysis. The GSEA output included enrichment scores, normalized enrichment scores (NES), nominal *p* values, FDR values, and leading-edge genes. For each predefined module, the expression of each gene was z-scored across patients, and the module score was calculated as the average z-score of the detected module genes. The NIS-high and NIS-low groups were compared using Welch-style tests followed by Benjamini–Hochberg FDR correction. The resulting scores are referred to as custom module scores or GSVA-like scores.

### 4.6. Immune Checkpoint Gene Analysis

Predefined immune checkpoint genes, including PDCD1, CD274, CTLA4, LAG3, TIGIT, HAVCR2, PDCD1LG2, VSIR, and IDO1, were compared between NIS-high and NIS-low tumors using the same patient-level expression data and statistical framework described for the differential expression analysis.

### 4.7. GEO Cohort Selection and Data Processing

Independent GEO colorectal cancer cohorts included GSE39582, GSE17536, GSE17537, and GSE14333 were used to externally validate the prognostic value of NIS. Gene expression data were obtained from curated CRC Data Bioconductor package, which compiles colorectal cancer transcriptomic datasets from public repositories, including the GEO. Uniformly processed fRMA gene-level ExpressionSet objects were used whenever available to reduce variability arising from differences in probe annotation and preprocessing across microarray platforms. The external validation cohort GSE39582 was used as the main validation cohort for DFS/RFS. GSE17536, GSE17537, and GSE14333 were used as additional validation cohorts according to the availability of OS and DFS/RFS endpoints.

### 4.8. Reconstruction of NIS in GEO Cohorts

NIS was reconstructed independently within each GEO cohort. For each dataset, the gene expression matrix was first gene-wise-standardized using z-score transformation: z = (x − mean[x])/SD[x], where x represents the expression value of a given gene across samples within the same cohort.

The neural/stromal score was calculated as the mean z-score of available neural/stromal module genes, and the immune activation score was calculated as the mean z-score of available immune activation module genes. NIS was then defined as NIS = neural/stromal score − immune activation score. Patients were divided into NIS-low and NIS-high groups using the median NIS within each cohort. Gene coverage was assessed for each dataset to confirm the availability of NIS component genes.

### 4.9. Biological Signature Analysis in GEO Cohorts

To assess whether the biological features associated with NIS were preserved in external datasets, predefined gene signatures were evaluated in each GEO cohort. These signatures included neural/stromal programs, cytotoxic T/NK cell activity, antigen presentation, immune checkpoint-related genes, CAF/fibroblast programs, extracellular matrix remodeling, EMT, and TGF-β signaling. For each signature, a sample-level score was calculated as the mean z-score of available genes in the signature. Signature scores were compared between NIS-high and NIS-low groups. Multiple testing was controlled using the Benjamini–Hochberg false discovery rate method.

### 4.10. Multi-Cohort Meta-Analysis

A multi-cohort meta-analysis was performed to evaluate the prognostic association of NIS across TCGA and GEO cohorts. Univariable Cox proportional-hazards regression estimates comparing the NIS-high and NIS-low groups were used as effect sizes. Hazard ratios (HRs) were log-transformed, and their corresponding standard errors (SEs) were calculated from the reported 95% confidence intervals (CIs) as follows:SE = [ln(upper CI) − ln(lower CI)]/3.92.

Random-effects meta-analysis was performed using the DerSimonian–Laird method. Separate analyses were conducted for OS and DFS/RFS endpoints and constituted the primary endpoint-specific meta-analyses. Between-study heterogeneity was evaluated using Cochran’s Q statistic, the I^2^ statistic, and the between-study variance (τ^2^).

### 4.11. Comparison with Established Transcriptomic Signatures

To evaluate whether NIS provides information beyond existing transcriptomic biomarkers, we performed a comparator analysis in TCGA-CRC. Using the same expression matrix employed for NIS construction, predefined scores representing immune infiltration, stromal enrichment, EMT, CAF/ECM remodeling, and cytotoxic T/NK activity were calculated as the mean z-score of their respective module genes. Because the original pipeline did not implement formal ESTIMATE or consensus CMS classifiers, we report these as ESTIMATE-like immune/stromal scores and exploratory CMS-like subtypes; CMS-like subtype was assigned by the highest of four literature-aligned signature scores (immune/MSI-like, canonical epithelial/WNT-like, metabolic-like, and mesenchymal/stromal-like). Prognostic performance was assessed by univariable and adjusted (age, sex, stage) Cox models, Harrell’s C-index, time-dependent AUC, likelihood-ratio improvement, and ΔC-index relative to the clinical model. Complementarity was evaluated by Spearman correlation between NIS and comparator scores, NIS distribution across CMS-like groups, NIS stratification within the CMS4-like subgroup, and identification of stromal-high/immune-low tumors. Detailed results are provided in Table S11.

### 4.12. Single-Cell Validation and Focused Ligand–Receptor Analysis

Single-cell analysis was performed using the author-processed colorectal cancer scRNA‑seq dataset from Lee et al. (2020) [29] (GSE132465), downloaded as an h5ad file from Zenodo (doi:10.5281/zenodo.3967538). Patient, sample, tissue status, major cell type, fine cell annotation, quality-control metrics, and UMAP coordinates were extracted directly from the h5ad object. All UMAP visualizations were generated using the author-provided coordinates.

For NIS scoring, raw sparse counts were extracted from h5ad raw/X matrix using a low-memory h5py row-slicing workflow. Only predefined NIS genes and selected marker genes were extracted, and gene coverage was audited before scoring. For each cell, counts were normalized to counts per 10,000 using the h5ad-provided n_counts value and log1p-transformed. Each detected gene was subsequently z-score-standardized across all cells. The cell-level NC and IA scores were calculated as the mean standardized expression of the detected genes in the corresponding modules, and single-cell NIS was defined as NC score minus IA score. Cell-level scores were summarized by tissue, major cell type, fine cell annotation, patient, and sample.

Focused ligand–receptor (LR) analysis was performed as a hypothesis-driven, expression-based evaluation of predefined candidate axes rather than as a comprehensive cell–cell communication inference workflow. The candidate panel included LR axes related to immune checkpoint, TGF-β, chemokine, SPP1, ECM–integrin, angiogenic, PDGF, neurotrophic, neuregulin, and axon guidance. Raw counts for the selected ligand and receptor genes were extracted from the GSE132465 h5ad raw sparse matrix and normalized as log1p(counts per 10,000), after which mean expression was calculated by tissue and cell annotation. For each source-target cell-type pair, an expression-based LR score was calculated as LR score = mean normalized ligand expression in source cells × mean normalized receptor expression in target cells. NIS-relevant source populations included glial cells, fibroblasts, pericytes, endothelial cells, colorectal cancer cells, and selected myeloid/dendritic populations. Target populations included cytotoxic, effector memory, exhausted-like, and proliferating CD8 T cells, regulatory T cells, NK cells, myeloid/dendritic cells, and colorectal cancer cells. Tumor-versus-normal differential communication was calculated as the tumor LR score minus the normal LR score for matched source-target major cell-type pairs. These expression-based scores were used to prioritize candidate LR axes for biological interpretation rather than to establish direct or causal cell–cell communication.

All single-cell analyses were performed in Python 3.11.15 using NumPy 2.4.6, pandas 3.0.5, SciPy 1.17.1, h5py 3.16.0, Matplotlib 3.11.1, and openpyxl 3.1.5. The h5ad object was accessed directly through h5py; therefore, Scanpy and AnnData were not used for data processing. The described workflow contained no stochastic processing steps, and no random seed was required. A complete software-version record is provided in Table S10.

### 4.13. Spatial Transcriptomics Analysis of NIS

Processed colorectal cancer spatial transcriptomics h5ad files generated by *Valdeolivas* et al. [30] were used to assess the spatial localization of NIS. A total of 12 spatial transcriptomic slides (17,326 spots) with complete ancillary data (expression matrix, coordinates, annotations, and cell-type labels) were sourced from the local repository. This convenience sample was not randomized nor preselected for NIS, patient outcomes, or immune/stromal abundance, and thus reflects a data-availability cohort rather than a biologically stratified one. For each slide, NC and IA signature genes were extracted, and NIS was calculated independently within each slide to minimize the effects of inter-section differences in sequencing depth and technical variation. Raw counts of target genes were normalized as counts per 10,000 per spot and log1p-transformed. Gene-wise z-scores were subsequently calculated across spots within each section. For each spot, the NC score and IA score were calculated as the mean z-score of detected genes belonging to the corresponding signatures.

The spot-level NIS was defined as NIS = NC score − IA score.Spot-level NIS values were summarized by pathologist annotation, deconvolved spatial cell-type label, sample identity, and sample-region combination. For each group, the number of spots, mean NC score, mean IA score, mean and median NIS, and median total counts were calculated. Gene coverage for NIS modules was audited by sample and module.

To characterize the molecular features associated with NIS-defined spatial niches, Pearson and Spearman correlation analyses were performed between spot-level NIS and representative marker genes, including neural/glial, stromal/ECM, cytotoxic T-cell, antigen-presentation, and immune checkpoint markers.

To provide orthogonal protein-level support, we audited public IHC and protein-localization data for a 13-marker panel (neural/glial, stromal/ECM, cytotoxic immune, and checkpoint axes) using the Human Protein Atlas and published CRC studies (Table S9). Evidence was graded as strong, moderate, weak/indirect, or unavailable and was regarded as public supportive evidence, not cohort-specific experimental validation.

### 4.14. Statistical Analysis

Kaplan–Meier curves and log-rank tests were used for survival visualization and between-group comparisons. Cox proportional-hazards models were employed to estimate hazard ratios and 95% confidence intervals. Multivariable Cox models were adjusted for available clinicopathological covariates, primarily age, sex, and pathological stage. Multiple testing correction was performed using the Benjamini–Hochberg method.

## 5. Conclusions

In summary, this study identifies NIS as an interpretable neuro-immune microenvironment score associated with adverse outcomes in CRC. Multi-layered validation suggests that NIS-high tumors are characterized by neural/stromal and ECM remodeling, vascular/pericyte-associated niches, myeloid and stromal immune regulation, and reduced or spatially separated cytotoxic immune activation. These findings provide a framework for understanding how neural–stromal remodeling and immune imbalance may cooperate in CRC progression and generate hypotheses for future biomarker-driven and microenvironment-targeted therapeutic studies.

## Figures and Tables

**Table 1:** Study cohorts and analytic datasets used to construct, validate and mechanistically interpret NIS.

Analysis Block	Dataset/Cohort	Role in Manuscript	Analyzed Samples/Patients	Endpoint or Readout	NIS Gene Coverage	Key Manuscript Note
TCGA discovery and internal clinical analysis	TCGA-COAD + TCGA-READ	Discovery cohort for NIS construction, clinical association, OS analysis and transcriptomic mechanism	624	Overall survival; NIS-high versus NIS-low; transcriptomic programs	NC 21/21; IA 28/28	Primary analysis used total CRC cohort
GEO external validation	GSE39582	Primary external recurrence/death validation	557 DFS/RFS-evaluable patients	DFS/RFS	NC 21/21; IA 27/28	Strongest external validation dataset; do not describe as OS validation
GEO external validation	GSE17536	External/supplemental validation	177 OS-evaluable; 127 DFS/RFS-evaluable patients	OS and DFS/RFS	NC 21/21; IA 27/28	OS significant; DFS/RFS directionally consistent but underpowered/non-significant
GEO external validation	GSE17537 and GSE14333	Additional directionality/sensitivity cohorts	GSE17537: 55 OS and 50 DFS/RFS; GSE14333: 226 DFS/RFS	OS/DFS/RFS where available	NC 21/21; IA 27/28	Directionally consistent estimates; limited by event counts or unavailable OS
Single-cell mechanism	GSE132465/Lee et al. CRC scRNA-seq processed h5ad	Cellular localization and mechanism support	61,370 cells; 23 patients; 33 samples	Cell-type and fine-state NIS; focused ligand–receptor axes	NC 21/21; IA 28/28	Mechanistic localization; not patient-level prognostic validation
Spatial transcriptomics support	Valdeolivas-derived CRC spatial transcriptomics processed h5ad subset	Spatial localization and orthogonal support	12 slides; 17,326 spots	Spot-level NIS by pathology region, ST-cell-type label and marker correlation	NC 21/21; IA 28/28	Local processed subset, not the full originally reported 14-slide dataset

**Table 2:** Primary prognostic associations and external validation summary for NIS-high versus NIS-low.

Cohort/Analysis	Endpoint	*n*/Events	Model	Effect Estimate for NIS-High vs. NIS-Low	Adjusted Covariates	Interpretation for Manuscript
TCGA-COAD/READ	OS	620/129	Univariable Cox	1.46 (1.02–2.08); *p* = 0.0377	None	NIS-high was associated with worse OS in the discovery cohort
TCGA-COAD/READ	OS	501/83	Multivariable Cox	1.23 (0.79–1.91); *p* = 0.357	Age, sex and pathological stage	Direction remained concordant but was attenuated and not statistically significant after clinical adjustment
GSE39582	DFS/RFS	557/177	Univariable Cox in external GEO cohort	1.74 (1.28–2.35); *p* < 0.001	Dataset-specific; multivariable estimates used only when covariates/events sufficient	External recurrence/death validation significant; high NIS associated with worse DFS/RFS
GSE17536	OS	177/73	Univariable Cox in external GEO cohort	1.91 (1.18–3.08); *p* = 0.00837	Dataset-specific; multivariable estimates used only when covariates/events sufficient	Directionally consistent with high-NIS worse prognosis; statistical strength depends on endpoint/sample size
GSE17536	DFS/RFS	127/36	Univariable Cox in external GEO cohort	1.44 (0.75–2.78); *p* = 0.273	Dataset-specific; multivariable estimates used only when covariates/events sufficient	Directionally consistent with high-NIS worse prognosis; statistical strength depends on endpoint/sample size
GSE17537	OS	55/20	Univariable Cox in external GEO cohort	1.28 (0.53–3.09); *p* = 0.584	Dataset-specific; multivariable estimates used only when covariates/events sufficient	Directionally consistent with high-NIS worse prognosis; statistical strength depends on endpoint/sample size
GSE17537	DFS/RFS	50/19	Univariable Cox in external GEO cohort	1.63 (0.64–4.18); *p* = 0.305	Dataset-specific; multivariable estimates used only when covariates/events sufficient	Directionally consistent with high-NIS worse prognosis; statistical strength depends on endpoint/sample size
GSE14333	DFS/RFS	226/50	Univariable Cox in external GEO cohort	1.31 (0.75–2.28); *p* = 0.347	Dataset-specific; multivariable estimates used only when covariates/events sufficient	Directionally consistent with high-NIS worse prognosis; statistical strength depends on endpoint/sample size
Random-effects meta-analysis (OS only)	OS only	See component cohorts	DerSimonian–Laird random-effects	1.57 (1.20–2.06); *p* = 0.00115; I^2^ = 0%	Pooled univariable endpoint estimates	Cross-cohort synthesis supported a consistent adverse prognostic direction for NIS-high
Random-effects meta-analysis (DFS/RFS only)	DFS/RFS only	See component cohorts	DerSimonian–Laird random-effects	1.60 (1.26–2.03); *p* < 0.001; I^2^ = 0%	Pooled univariable endpoint estimates	Cross-cohort synthesis supported a consistent adverse prognostic direction for NIS-high
Random-effects meta-analysis (all prognostic endpoints)	All prognostic endpoints	See component cohorts	DerSimonian–Laird random-effects	1.59 (1.33–1.90); *p* < 0.001; I^2^ = 0%	Pooled univariable endpoint estimates	Cross-cohort synthesis supported a consistent adverse prognostic direction for NIS-high

## Data Availability

Bulk transcriptomic data were obtained from TCGA and GEO (GSE39582, GSE17536, GSE17537, and GSE14333). The single-cell RNA-seq dataset is available from GEO under accession GSE132465, and the processed spatial transcriptomics data are available from Zenodo (record 7760264). Analysis code for NIS construction, validation, and downstream analyses is publicly available at https://github.com/huhui1993/NIS_CRC (accessed on 3 August 2026). Additional supporting data are provided in the article and Supplementary Materials.

## Supplementary Materials

The following supporting information can be downloaded at https://www.mdpi.com/article/10.3390/ijms27167231/s1.
